# Case Report—Uterine Necrosis: A Rare Complication of Uterine Artery Embolization in Postpartum Hemorrhage

**DOI:** 10.3390/reports9020167

**Published:** 2026-05-24

**Authors:** Soobin Lee, Nari Kim, Myung Shin Shin, Haeyoun Kang, Sang Hee Jung

**Affiliations:** 1 Department of Obstetrics and Gynecology, CHA Ilsan Medical Center, CHA University School of Medicine, Ilsan, Goyang 10414, Republic of Korea; 2 Department of Obstetrics and Gynecology, CHA Bundang Medical Center, CHA University School of Medicine, Bundang, Seongnam 13496, Republic of Korea; 3 Foryou Moon OBGY Clinic, Seoul 05551, Republic of Korea; 4 Department of Pathology, CHA Bundang Medical Center, CHA University School of Medicine, Bundang, Seongnam 13496, Republic of Korea

**Keywords:** abdominal hysterectomy, pelvic arteriography, postpartum hemorrhage, uterine artery embolization, uterine necrosis

## Abstract

**Background and Clinical Significance:** Postpartum hemorrhage (PPH) is a leading cause of maternal mortality worldwide. Among its various etiologies, uterine atony accounts for approximately 70% of cases, while other causes include genital tract trauma, pathologic placentation, and intrapelvic arterial injury. Uterine artery embolization (UAE) has emerged as a preferred management option for severe PPH due to its high success rates of 89–98% and fertility preservation benefit. Despite its efficacy, UAE can lead to complications, such as pain, re-bleeding, infection, persistent vaginal discharge, ovarian insufficiency, and uterine necrosis—a rare but serious complication occurring in 1.4–2.7% of cases. **Case Presentation:** We present three cases of uterine necrosis following UAE from a single center (CHA Bundang Medical Center) between 2003 and 2024. All patients developed persistent high-grade fever approximately two weeks after the procedure, despite an initial response to antibiotic therapy. Imaging studies, including contrast-enhanced CT and MRI, revealed uterine ischemia and necrosis, and all patients ultimately required total hysterectomy. **Conclusions:** Uterine necrosis is a rare but potentially life-threatening complication of UAE that should be suspected in patients with persistent high-grade fever beyond the typical post-procedural course. Early imaging evaluation, particularly with contrast-enhanced modalities, is essential for prompt diagnosis. Timely surgical intervention, including hysterectomy, may be required to prevent severe morbidity.

## 1. Introduction and Clinical Significance

Postpartum hemorrhage (PPH), a leading cause of maternal mortality, affects approximately 14 million women globally each year, causing around 70,000 deaths [1]. PPH can result from uterine atony, genital tract trauma, pathologic placentation, or intrapelvic arterial injury [2]. Managing PPH involves restoring intravascular volume, with interventions focusing on uterine contraction since uterine atony accounts for nearly 70% of cases [3]. According to the guidelines recommended by the Federation of Gynecology and Obstetrics (FIGO) and the World Health Organization (WHO), initial management includes uterine massage, bimanual compression, and administration of uterotonic agents such as oxytocin, sulprostone, carbetocin and misoprostol [4]. When conservative measures fail, surgical methods, such as B-lynch sutures, may also be necessary [2]. In cases of uncontrollable PPH, despite these interventions, hysterectomy has traditionally been performed to decrease maternal mortality. With the development of interventional radiology, transcatheter uterine arterial embolization (UAE) has become a preferred alternative for those desiring to preserve future fertility [5].

UAE temporarily blocks uterine arteries with biocompatible particles to control bleeding [6]. In severe PPH, it offers many advantages over hysterectomy, including repeatability, minimally invasive nature, and preservation of the uterus and fertility [6]. Previous studies demonstrated a high success rate for UAE, ranging from 89% to 98%, making it an essential tool in PPH management [6,7]. However, complications can occur including pain, re-bleeding, infection, persistent vaginal discharge, ovarian insufficiency, and uterine necrosis [5].

Uterine necrosis, although exceedingly rare, occurring in 1.4% to 2.7% of cases, is a serious complication that can lead to sepsis and often necessitates hysterectomy [8,9]. Poujade et al. identified only 19 reported cases of uterine necrosis following UAE for PPH between 1985 and 2013, with a mean time interval of 21 days (range 9–730 days) between embolization and diagnosis [9]. At CHA Bundang Medical Center, a multidisciplinary maternal–fetal care center in South Korea, only three cases of uterine necrosis were reported among 313 UAE procedures for PPH from 2003 to 2024, indicating a complication rate of less than 1%. Herein, we present three cases of uterine necrosis following UAE, detailing the clinical course, along with a brief literature review and potential management options (Table 1).

## 2. Case Presentation

Case 1

A 39-year-old woman with a singleton pregnancy (gravida2 para0; G2P0), body mass index (BMI) of 24.2 kg/m^2^, presented to the emergency room (ER) at 34 weeks of gestation due to preterm premature rupture of membrane (PPROM) with breech presentation. She had no significant medical history or smoking history. Her only prior surgical history was dilation and curettage (D&C) for missed abortion performed 3 years earlier. She had severe adenomyosis, and multiple myomas managed conservatively. During the cesarean section, significant bleeding (approximately 1000 mL) and placenta accreta were noted. Despite uterine massage, administration of uterotonics, and surgical hemostatic sutures, bleeding control was unsuccessful.

Arteriography revealed diffuse hypertrophy in both uterine arteries leading to emergency bilateral UAE using gelatin sponge particles (Cali-Gel 560–1400 μm; Alicon, Hangzhou, China) mixed with contrast media (Figure 1). After embolization, vaginal bleeding had stopped, and the patient’s vital signs stabilized. However, the next day, she developed a fever (38.1 °C) with abdominal discomfort. Blood assessment, cervical culture, and sonography revealed no abnormalities except for elevated C-reactive protein (CRP), peaking up to 20.9 mg/dL. The patient was treated with antibiotics, including ceftriaxone, metronidazole, and clarithromycin, for 10 days, resolving the fever and lowering CRP levels, leading to the patient’s discharge.

On the 16th day post-discharge, she was readmitted with a recurrent fever of up to 38.5 °C. Extensive antibiotic treatment with teicoplanin and meropenem was administered, and a contrast-enhanced magnetic resonance imaging (CE-MRI) revealed a substantial uterine hematoma with necrosis. Despite continuous antibiotics, fever and abdominal pain persisted with high CRP level of 20.65 mg/dL. The patient had no plan for future fertility; thus, a total hysterectomy was performed. Grossly, the surgery revealed near-total uterine infarction. Pathologically, acute inflammation of endometrium and myometrium with extensive coagulative necrosis was confirmed. The patient’s subsequent progress was favorable.

Case 2

A 39-year-old woman, conceived via in vitro fertilization (IVF), with a singleton pregnancy (gravida 1, para 0; G1P0) and a BMI of 26.2 kg/m^2^, had no smoking history and no significant past medical or surgical history, except for adenomyosis and multiple myomas managed conservatively. After spontaneous vaginal delivery at 39 weeks of gestation, she encountered severe uterine bleeding, with estimated blood loss of 1800 mL due to poor uterine contraction. Despite administration of uterotonics, uterine bleeding was uncontrolled. Pelvic arteriogram revealed hypertrophied bilateral uterine arteries, leading to bilateral UAE with 560–1400 μm sized Cali-Gel gelatin sponge particles. After UAE, uterine bleeding stopped, but the patient presented high fever (>38.5 °C) with abdominal discomfort on the third day of post-UAE. CRP was elevated up to 19.43 mg/dL, but blood and cervical culture revealed no specific growth. Antibiotics including piperacillin-tazobactam with teicoplanin were administered in order to suppress inflammation, although the definite origin of the high fever was not identified. After one week of antibiotic treatment, her fever subsided and she was discharged.

On the 15th day after UAE, the patient visited the ER with recurrence of high fever (38.7 °C). An abdominal–pelvic contrast-enhanced CT (CECT) revealed a large hematoma in the endometrial cavity (Figure 2), suspected to be the cause of the fever. Thus, D&C was performed to remove hematoma and resolve fever. However, within two weeks, fever (38 °C) and severe abdominal pain recurred. A follow-up abdominal–pelvic CECT revealed persistent hematoma, leading to a second D&C. Extended necrosis and inflammation near the entire uterine myometrium raised suspicion of total uterine necrosis. After consulting the patient, a total hysterectomy was decided. Total hysterectomy was performed showing diffuse coagulative necrosis with total infarction, and the patient’s subsequent progress was favorable.

Case 3

A 31-year-old woman with a singleton pregnancy (gravida 1, para 0; G1P0) and a BMI of 17.9 kg/m^2^, with no smoking history and no significant past medical or surgical history, was transferred to the ER for UAE due to postpartum bleeding after cesarean section at 39 weeks of gestation. Prior to transfer, a B-Lynch Suture had already been placed during the cesarean section at the referring hospital as an initial surgical intervention for postpartum hemorrhage. Uterine arteriogram showed right uterine artery hypertrophy and multifocal pseudo-aneurysm at the left uterine artery branch. Bilateral UAE was performed using glue (Histoacryl; B.Braun, Aesculap AG, Tuttlingen, Germany) and gelatin sponge particles (Cali-Gel 1000–1400 μm; Alicon, Hangzhou, China). The bleeding stopped, but on the second day after UAE, she started to develop a high fever (38.8 °C) with generalized abdominal pain.

Despite prior antibiotic treatment, her symptoms persisted, and the CRP level increased to 9.35 mg/dL. On the 7th day, abdominal–pelvic CECT revealed a large uterine hematoma, suspected to be the cause of inflammation. D&C was performed to remove the hematoma, revealing infarcted myometrium tissue with acute inflammatory exudate, raising suspicions of uterine necrosis. Despite the D&C, symptoms including high fever with consistent abdominal pain persisted. Due to concerns of sepsis, a total hysterectomy and bilateral salpingectomy were performed on the 15th day. The hysterectomy specimen showed a fragile uterus with severe necrosis and inflammation above the previously placed B-lynch suture. Histopathology confirmed chronic focal endometritis and near-total myometrial infarction with abscess (Figure 3). Postoperatively, the patient fully recovered without any further obstetrical or gynecological problems.

## 3. Discussion

Uterine artery embolization (UAE) has been widely known as a fertility-preserving intervention for postpartum hemorrhage. However, in all of our three cases, the patient ultimately required hysterectomy due to progressive uterine necrosis. These findings suggest that the fertility-preserving benefit of UAE may not always be achieved in clinical practice when severe ischemic complications occur.

Uterine necrosis after UAE manifests in various ways and times, with its exact pathophysiology remaining unknown. Nonetheless, several hypotheses exist based on the literature reviewed.

Firstly, the size and type of embolic agents used in UAE can contribute to uterine necrosis. Small particles (<500 μm) can occlude distal collateral vessels, particularly postpartum when the distended uterus allows small particles to reach more distant vessels than usual, possibly leading to insufficient blood supply and subsequent uterine necrosis [10,11]. Thus, particles larger than 500–700 μm are recommended in embolization for PPH to minimize the risk of distal vessel occlusion [9]. Additionally, PVA particles tend to aggregate, blocking the microcatheter and proximal vessels [12]. In contrast, gelatin sponge is preferred due to its efficiency, safety, and availability. It typically dissolves within 3–6 weeks, allowing recanalization of the targeted embolized artery, which reduces the risk of long-term ischemia and future infertility [13].

Anatomic factors may also contribute to uterine necrosis. It is well established that collateral arteries from the cervicovaginal artery, ovarian artery, round ligament artery, and internal pudendal artery often participate in a complex anastomotic network, supplying blood to the uterus and serving as potential bleeding source in PPH [13]. The absence of this anastomotic network, whether due to congenital or acquired reasons, may elevate the risk of uterine necrosis through poor blood supply when both uterine arteries are embolized [14]. Finally, various other risk factors, including advanced age, a history of radiation therapy, lack of antibiotic prophylaxis, sepsis, massive bleeding, hypovolemic shock, and a history of cocaine abuse, can contribute to the likelihood of uterine necrosis [9,14].

All three cases we present followed the current guidelines, using absorbable gelatin particles larger than 560 μm during UAE [13]. In the third case, however, Histoacryl (N-butyl-2-cyanoacrylate), a permanent liquid embolic agent unlike gelatin particles, was additionally used. Compared with gelatin sponge alone, the combination of a liquid embolic agent and gelatin sponge may result in different occlusion characteristics, including more permanent vascular occlusion. However, the procedural rationale for selecting this combination could not be retrospectively confirmed, and its specific indication in this case remains unclear. Also, to our knowledge, no previous studies have suggested that this agent contributes to uterine necrosis [15,16]. Although N-butyl-2-cyanoacrylate is more commonly utilized in gastric variceal embolization than in uterine embolization, reported embolic complications in those procedures mainly involve distant glue embolization, with an incidence of 0.5–4.3% in a 25-year retrospective review [17]. Importantly, no evidence currently supports a causal relationship between this agent and uterine necrosis, and its clinical impact in uterine embolization remains unclear. Nevertheless, a definitive relationship cannot be entirely excluded due to the limited number of reported cases. Also, none of the three cases examined had any history of radiation therapy or cocaine abuse. Although massive bleeding did occur, it did not reach the extent of hypovolemic shock, as active management was promptly undertaken during PPH.

One of the identifiable risk factors was advanced maternal age (39 years) in Cases 1 and 2. The anastomotic vascular network of the uterus in all three cases was not specifically assessed prior to UAE. Consequently, the adequacy of collateral perfusion cannot be conclusively determined in these patients.

Adenomyosis and uterine myomas were present in Cases 1 and 2, respectively. Although these benign uterine conditions are not established independent risk factors for uterine necrosis following uterine artery embolization, they may act as confounding factors by altering uterine vascular architecture or local perfusion. Nevertheless, their direct contribution to uterine necrosis in our cases remains uncertain. Taken together, our cases suggest that uterine necrosis after UAE is likely multifactorial, involving procedural factors, patient-specific vascular anatomy, and underlying clinical conditions.

According to the clinical perspectives, all three cases exhibit high-grade fever over 38.0 °C after UAE, which initially resolved with antibiotic treatment but recurred within two weeks. This is consistent with the finding from Poujade’s review, where symptoms appeared between 3 and 150 days post-procedure, with a mean interval of 21 days [9]. Our cases demonstrated symptom onset at 16, 15, and 15 days, respectively, falling within the typical timeframe.

In our institutional retrospective review, fever over 38.0 °C following UAE was observed in 53% of cases among 313 UAE procedures for PPH, indicating that fever itself is a relatively common post-procedural symptom. However, the duration of fever was short, with a mean duration of 1.73 days and a mean peak temperature of 38.4 °C. Therefore, persistent high-grade fever should not be disregarded and warrants further investigation. Because persistent fever after UAE may result from various conditions, including endometritis, septic pelvic thrombophlebitis, retained products of conception, pelvic abscess, or post-embolization syndrome, proactive imaging evaluation with contrast-enhanced CT or MRI should be considered for accurate differential diagnosis and timely detection of serious complications. In Cases 2 and 3, D&C was performed because persistent fever raised the possibility of alternative intrauterine causes, such as endometritis or infected intrauterine hematoma. However, when uterine necrosis is present, D&C should be performed with caution because necrotic and friable myometrial tissue may increase the risk of uterine perforation. In addition, delayed recognition of uterine necrosis may potentially postpone definitive surgical management, including hysterectomy.

CE-MRI is particularly valuable for its precision in visualizing soft tissues such as the uterine myometrium and endometrium. Diagnostic features of uterine necrosis include an enlarged uterus with diffusely hypo-perfused myometrium with air bubbles, along with an irregular and blurred demarcation between the endometrium and myometrium [9]. These imaging findings were confirmed in our cases, where contrast-enhanced CE-MRI and CECT demonstrated large uterine hematomas with necrotic tissue.

This study has several limitations that should be considered when interpreting our findings. First, although UAE has been widely regarded as an intervention for PPH, all patients in our series ultimately required hysterectomy due to progressive uterine necrosis. Therefore, our findings may not fully represent the overall fertility-preserving potential of UAE, but rather highlight the possibility of rare yet severe ischemic complications in selected patients.

Second, all three patients underwent bilateral embolization, which remains the standard hemostatic approach for severe postpartum hemorrhage at our institution. While bilateral embolization may theoretically increase the risk of uterine ischemia by reducing collateral perfusion, its independent contribution to uterine necrosis could not be definitively determined in this study. In addition, no direct comparison was available with alternative surgical approaches such as internal iliac artery ligation, which have been reported to preserve ovarian function and uterine perfusion [18,19].

Finally, the retrospective nature and small sample size of this case series inevitably limit the generalizability of our findings. Further comparative studies are needed to better identify optimal hemorrhage-control strategies while minimizing ischemic complications.

## 4. Conclusions

In conclusion, our cases suggest that uterine necrosis following uterine artery embolization (UAE) is likely a multifactorial complication involving procedural factors, patient-specific vascular anatomy, and underlying clinical conditions. Despite adherence to current embolization recommendations using absorbable particles larger than 560 μm, severe ischemic complications still occurred and ultimately resulted in hysterectomy in all three patients. These findings highlight that although UAE is widely regarded as a fertility-preserving intervention for postpartum hemorrhage, its clinical benefits may be limited when irreversible uterine ischemia develops.

Persistent high-grade fever after UAE, particularly when recurring after initial improvement, should prompt early radiologic evaluation for possible uterine necrosis. Although no definite treatment guideline currently exists for uterine necrosis after UAE, total hysterectomy remains an effective treatment once progressive necrosis is diagnosed, preventing further infectious and systemic complications. Future studies should focus on identifying additional risk factors, optimizing treatment selection, and developing strategies to predict and prevent this rare but serious complication.

## Figures and Tables

**Figure 1:**
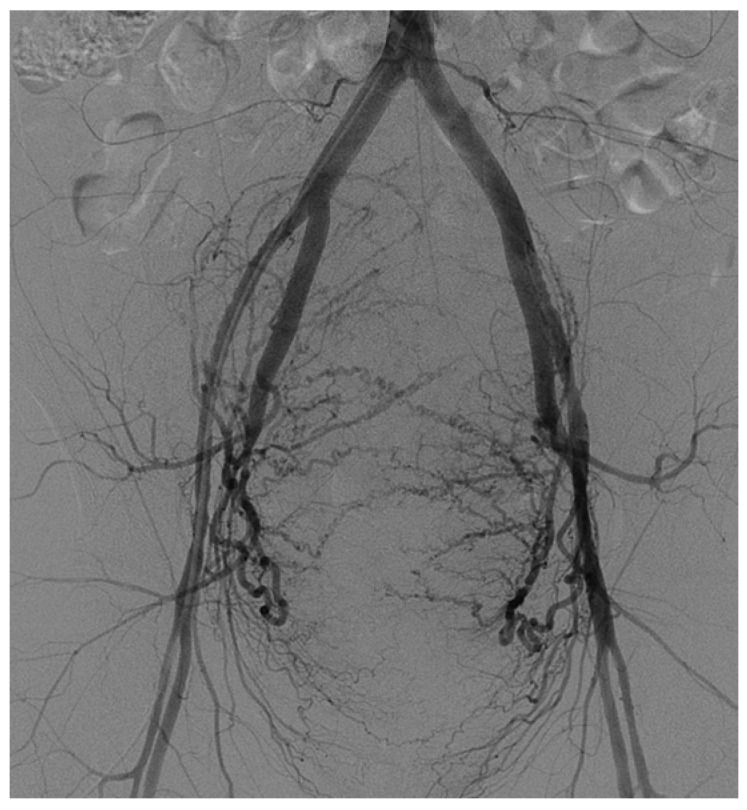
Pelvic arteriogram before uterine artery embolization. This shows diffuse hypertrophied bilateral uterine arteries with hypervascularity of enlarged uterus.

**Figure 2:**
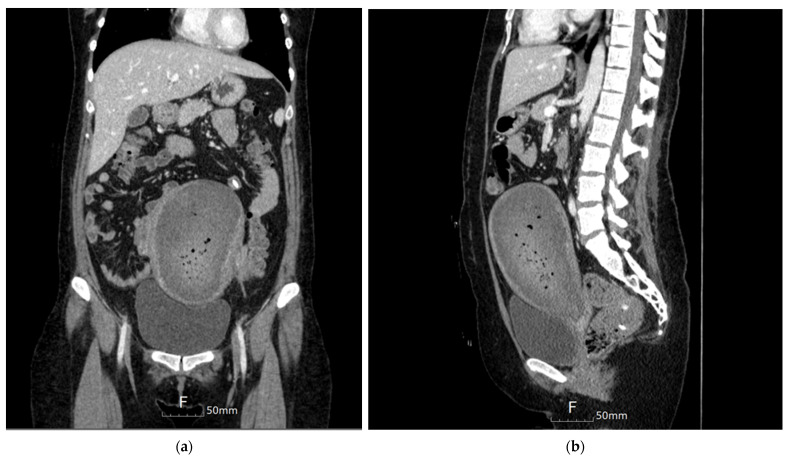
Contrast-enhanced CT of the abdomen and pelvis (CECT): (**a**) coronal View and (**b**) sagittal view of abdomen shows a large amount of air containing a hematoma in the endometrial cavity.

**Figure 3:**
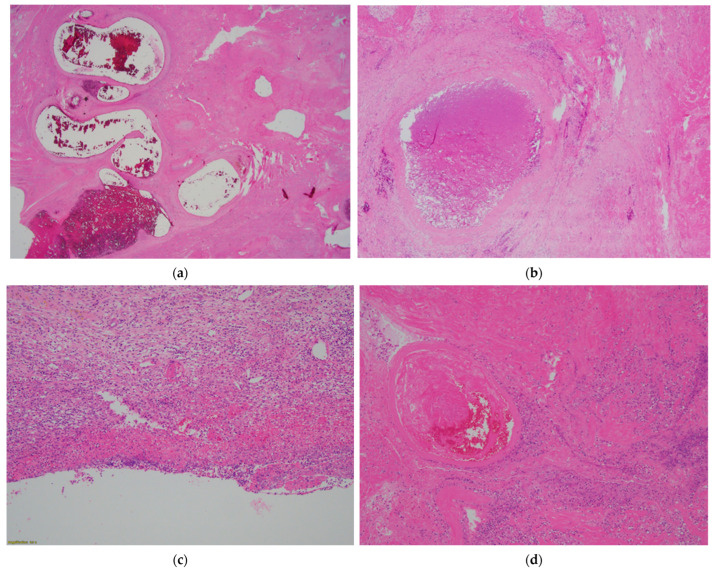
Representative images of the uterus. (**a**) There was diffuse congestion within myometrial vessels of varying sizes. H&E, ×12.5. (**b**) A thrombus was identified filling a relatively large blood vessel. H&E, ×40. (**c**) The endometrium had thinned, the glands were lost, and acute suppurative inflammation and hemorrhage took their place. H&E, ×100. (**d**) There was a thrombus within a sizable vessel, with vascular wall lysis and inflammation in the surrounding myometrium. H&E, ×100.

**Table 1:** Clinical timeline of the three patients with uterine necrosis following uterine artery embolization for postpartum hemorrhage.

Clinical Course	Case 1	Case 2	Case 3
**Maternal age**	39 years	39 years	31 years
**Obstetric history**	G2P0	G1P0	G1P0
**BMI**	24.2 kg/m^2^	26.2 kg/m^2^	17.9 kg/m^2^
**Delivery**	Cesarean section (34 weeks)	Vaginal Delivery (39 weeks)	Cesarean section (39 weeks)
**UAE**	Day 0	Day 0	Day 0
**Embolic material**	Gelatin sponge (>560 μm)	Gelatin sponge (>560 μm)	Gelatin sponge (>560 μm) +Histoacryl (>560 μm)
**Persistent or** **recurrent Fever** **(>38 °C)**	Recurrent Fever, (post-UAE day 16)	Recurrent Fever (post-UAE day 15)	Persistent fever (post-UAE day 2)
**D&C**	No	Yes (at post-UAE day 17 and day 30)	Yes (at post-UAE day 7)
**Imaging** **evaluation**	CE-MRI (at post-UAE day 32)	CECT (at post-UAE day 17 and day 30)	CECT (at Post-UAE day 7)
**Definitive** **Surgery**	Total hysterectomy (at post-UAE day 34)	Total hysterectomy (at post-UAE day 32)	Total hysterectomy + bilateral salpingectomy (at post-UAE day 15)
**Final Pathology**	Uterine necrosis	Uterine necrosis	Near-total myometrial infarction with abscess
**Outcome**	Full Recovery	Full Recovery	Full Recovery

## Data Availability

The data presented in this study are not publicly available due to patient privacy and ethical restrictions, as the dataset contains potentially identifiable clinical information from a small number of patients. The study was approved by the Institutional Review Board of Bundang CHA Medical Center, and the IRB does not permit public sharing of individual-level data. Data are available from the corresponding author upon reasonable request, subject to approval by the IRB and in accordance with relevant data protection regulations.

## Abbreviations

The following abbreviations are used in this manuscript:

PPH	Postpartum hemorrhage
UAE	Uterine artery embolization
BMI	Body mass index
CECT	Contrast-enhanced computed tomography
CE-MRI	Contrast-enhanced magnetic resonance imaging
CRP	C-reactive protein
ER	Emergency room
PPROM	Preterm premature rupture of membrane
D&C	Dilatation and curettage
IVF	In vitro fertilization
FIGO	International Federation of Gynecology and Obstetrics
WHO	World Health Organization
PVA	Polyvinyl alcohol
